# Rhodopsin-associated retinal dystrophy: Disease mechanisms and therapeutic strategies

**DOI:** 10.3389/fnins.2023.1132179

**Published:** 2023-04-03

**Authors:** Fangyuan Zhen, Tongdan Zou, Ting Wang, Yongwei Zhou, Shuqian Dong, Houbin Zhang

**Affiliations:** ^1^Department of Ophthalmology, The First Affiliated Hospital of Zhengzhou University, Henan Provincial Ophthalmic Hospital, Zhengzhou, China; ^2^The Key Laboratory for Human Disease Gene Study of Sichuan Province and Institute of Laboratory Medicine, Sichuan Provincial People’s Hospital, University of Electronic Science and Technology of China, Chengdu, Sichuan, China; ^3^Research Unit for Blindness Prevention, Chinese Academy of Medical Sciences (2019RU026), Sichuan Academy of Medical Sciences and Sichuan Provincial People’s Hospital, Chengdu, Sichuan, China

**Keywords:** rhodopsin, retinitis pigmentosa, retinal degeneration, gene therapy, stem cell therapy

## Abstract

Rhodopsin is a light-sensitive G protein-coupled receptor that initiates the phototransduction cascade in rod photoreceptors. Mutations in the rhodopsin-encoding gene *RHO* are the leading cause of autosomal dominant retinitis pigmentosa (ADRP). To date, more than 200 mutations have been identified in *RHO*. The high allelic heterogeneity of *RHO* mutations suggests complicated pathogenic mechanisms. Here, we discuss representative *RHO* mutations as examples to briefly summarize the mechanisms underlying rhodopsin-related retinal dystrophy, which include but are not limited to endoplasmic reticulum stress and calcium ion dysregulation resulting from protein misfolding, mistrafficking, and malfunction. Based on recent advances in our understanding of disease mechanisms, various treatment methods, including adaptation, whole-eye electrical stimulation, and small molecular compounds, have been developed. Additionally, innovative therapeutic treatment strategies, such as antisense oligonucleotide therapy, gene therapy, optogenetic therapy, and stem cell therapy, have achieved promising outcomes in preclinical disease models of rhodopsin mutations. Successful translation of these treatment strategies may effectively ameliorate, prevent or rescue vision loss related to rhodopsin mutations.

## 1. Introduction

Photoreceptors are the cells in the retina where light signals are converted to neural visual signals through the phototransduction cascade. The human retina contains two types of photoreceptors: rods and cones (Molday and Moritz, 2015). Rods account for 95% of all photoreceptors, and cones account for the remaining 5% (Molday and Moritz, 2015).

Rods are distinct from cones in structure and function. Rods expressing rhodopsin that are highly sensitive to light are responsible for scotopic vision, whereas cones contain cone opsins and are responsible for photopic vision and color vision. The loss of rods, cones or both in the retina causes devastating vision disorders that are collectively called retinal degeneration. Retinitis pigmentosa (RP) is the most common inherited retinal disorder, affecting 1/3,000 to 1/4,000 individuals worldwide (Hartong et al., 2006; Zhang, 2016; Perea-Romero et al., 2021). RP affects rod cell function and peripheral vision at the beginning of the disease. The patients experience night blindness. As the disease progresses, it also affects cones and impairs central vision. The patients develop tunnel vision and may become completely blind at its advanced stage. To date, over 70 genes have been identified to cause nonsyndromic RP that does not affect other organs or tissues (RetNet^1^). RP can be inherited in an autosomal dominant, autosomal recessive, or X-linked manner. Although rare, RP can also be inherited in mitochondrial or digenic forms (Kim et al., 2012; Wu et al., 2014; Zhang, 2016; Wu et al., 2019). This review aims to summarize the progress in studies on the mechanism and therapy for retinal disorders related to rhodopsin mutations, including RP and CSNB.

In rod photoreceptors, rhodopsin, a visual pigment, is formed by the conjugation of 11-*cis*-retinaldehyde to opsin proteins. Rhodopsin belongs to the G protein-coupled receptor family, and its activation initiates the very first step of phototransduction in rod photoreceptors upon the absorption of photons. Rhodopsin is synthesized in the inner segment of the rod and then processed and transported to the outer segment (Athanasiou et al., 2018). The capture of the photon by the chromophore in rhodopsin causes the isomerization of 11-*cis*-retinaldehyde to all-trans-retinaldehyde and a conformational change in the protein, leading to the activation of the downstream phototransduction cascade, which occurs within the photoreceptor outer segment.

The *RHO* gene, encoding the opsin protein and mapped to the long arm of chromosome 3 at 3q22.1, consists of 5 exons. The open reading frame is composed of nucleotides encoding 348 amino acid residues with a calculated molecular weight of ~39 kDa (Rosenfeld et al., 1992; Athanasiou et al., 2018). Rhodopsin constitutes ~85% of the protein mass of rod outer segment plasma membranes (Palczewski, 2014). As the most abundant protein in photoreceptors, rhodopsin is densely packed on the disc membrane (Molday and Moritz, 2015). Atomic force microscopy (AFM) and cryo-electron tomography has revealed that rhodopsin is organized in the disc membrane *via* a four-tier hierarchy—monomers, dimers, rows of dimers, and row pairs—which is critical for outer segment morphogenesis (Fotiadis et al., 2003; Gunkel et al., 2015; Ploier et al., 2016). Approximately half of the surface of each disc is occupied by rhodopsin, with the remainder filled mostly with lipids, cholesterol, and less abundant proteins (Palczewski, 2014; Molday and Moritz, 2015; Athanasiou et al., 2017).

Rhodopsin is irreplaceable and vital in the process of vision; one small mistake in the process of gene transcription, translation, folding processing, or delivery to designated places may lead to vision damage. Mutations in rhodopsin are the most common cause of autosomal dominant retinitis pigmentosa (ADRP; Wilson and Wensel, 2003; Malanson and Lem, 2009; Ferrari et al., 2011; Athanasiou et al., 2017). While about 20–30% of RP cases are ADRP (Daiger et al., 2014), *RHO* mutations account for 30–40% of ADRP cases. Although uncommon, rhodopsin mutations may cause dominant congenital stationary night blindness (CSNB; Singhal et al., 2013) and recessive RP (Kumaramanickavel et al., 1994; Kartasasmita et al., 2011). Over 200 mutations in *RHO*, including over 170 missense and nonsense mutations, have been associated with RP^2^ (Table 1). These mutations, varying from point mutations, insertions, and deletions to complex rearrangements, impair rhodopsin functions, ultimately leading to RP or CSNB symptoms through a variety of mechanisms, some of which will be described in detail in the following paragraphs. While the mechanisms underlying rhodopsin-related retinal dystrophy have been systemically reviewed previously (Athanasiou et al., 2018), here, we focus more on well-studies mechanisms that have been utilized for the development of potential therapeutic approaches. This review also integrates and updates the information on pharmacological intervention, optogenetics, gene therapy and gene editing, and stem cell therapy for rhodopsin-related retinal disorders that were reviewed previously (Athanasiou et al., 2018; Ikelle et al., 2020; Meng et al., 2020; Ortega and Jastrzebska, 2021; Piri et al., 2021; Ortega and Jastrzebska, 2022). Many of these therapeutical approaches are not gene-specific and can be also applied to retinal degeneration caused by other gene mutations.

**Table 1:** Identified missense and nonsense mutations in rhodopsin.

Mutant	Codon change	a.a change	Codon number	Mutant	Codon change	a.a change	Codon number
T4K	ACR-AAA	Thr-Lys	4	G106W	GGG-TGG	Gly-Trp	106
P12R	CCC-CGC	Pro-Arg	12	G109R	GGA-AGA	Gly-Arg	109
N15S	AAT-AGT	Asn-Ser	15	C110R	TGC-CGC	Cys-Arg	110
T17M	ACG-ATG	Thr-Met	17	C110Y	TGC-TAC	Cys-Tyr	110
G18D	GGT-GRT	Gly-Asp	18	C110S	TGC-TCC	Cys-Ser	110
V20G	GTR-GGA	Val-Gly	20	C110F	TGC-TTC	Cys-Phe	110
R21C	CGC-TGC	Arg-Cys	21	E113K	GAG-AAG	Glu-Lys	113
P23H	CCC-CAC	Pro-His	23	G114D	GGC-GAC	Gly-Asp	114
P23L	CCC-CTC	Pro-Leu	23	G114V	GGC-GTC	Gly-Val	114
P23A	CCC-GCC	Pro-Ala	23	E122G	GAA-GGR	Glu-Gly	122
Q28H	CAG-CAC	Gln-His	28	L125R	CTG-CGG	Leu-Arg	125
Q28H	CAG-CAT	Gln-His	28	W126Ter	TGG-TGA	Trp-Term	126
Q28R	CAG-CGG	Gln-Arg	28	W126L	TGG-TTG	Trp-Leu	126
M39R	ATG-AGG	Met-Arg	39	S127F	TCC-TTC	Ser-Phe	127
L40R	CTG-CGG	Leu-Arg	40	L131P	CTG-CCG	Leu-Pro	131
M44T	ATG-ACG	Met-Thr	44	R135P	CGG-CCG	Arg-Pro	135
F45L	TTT-CTT	Phe-Leu	45	R135L	CGG-CTG	Arg-leu	135
L46R	CTG-CGG	Leu-Arg	46	R135G	CGG-GCG	Arg-Gly	135
L47R	CTG-CGG	Leu-Arg	47	R135W	CGG-TGG	Arg-Trp	135
G51R	GGC-CGC	Gly-Arg	51	Y136Ter	TAC-TAA	Tyr-Term	136
G51A	GGC-GCC	Gly-Ala	51	V137M	GTG-ATG	Val-Met	137
G51V	GGC-GTC	Gly-Val	51	C140S	TGT-TCT	Cys-Ser	140
F52V	TTC-GTC	Phe-Val	52	R147C	CGC-TGC	Arg-Cys	147
F52Y	TTC-TAC	Phe-Tyr	52	Q150K	GAG-AAG	Glu-Lys	150
P53R	CCC-CGC	Pro-Arg	53	T160T	ACC-ACA	Thr-Thr	160
F56Y	TTC-TAC	Phe-Tyr	56	W161R	TGG-CGG	Trp-Arg	161
L57R	CTC-CGC	Leu-Arg	57	W161Ter	TGG-TAG	Trp-Term	161
T58R	ACG-AGG	Thr-Arg	58	M163T	ATG-ACG	Met-Thr	163
T58M	ACG-ATG	Thr-Met	58	A164E	GCG-GAG	Ala-Glu	164
Y60Ter	TAC-TAA	Tyr-Ter	60	A164V	GCG-GTG	Ala-Val	164
Q64Ter	CAG-TAG	Gln-Ter	64	C167R	TGC-CGC	Cys-Arg	167
R69H	CGC-CAC	Arg-His	69	C167Y	TGC-TAC	Cys-Tyr	167
N78I	AAC-ATC	Asn-Ile	78	C167W	TGC-TGG	Cys-Trp	167
L79P	CTR-CCR	Leu-Pro	79	A169P	GCA-CCA	Ala-Pro	169
V87L	GTC-CTC	Val-Leu	87	P170H	CCC-CAC	Pro-His	170
V87D	GTC-GAC	Val-Asp	87	P170R	CCC-CGC	Pro-Arg	170
L88P	CTA-CCR	Leu-Pro	88	P171Q	CCA-CAA	Pro-Gln	171
G89R	GGT-CGT	Gly-Arg	89	P171L	CCA-CTA	Pro-Leu	171
G89D	GGT-GAT	Gly-Asp	89	P171S	CCA-TCA	Pro-Ser	171
G90D	GGC-GAC	Gly-Asp	90	G174S	GGC-AGC	Gly-Ser	174
G90V	GGC-GTC	Gly-Val	90	S176F	TCC-TTC	Ser-Phe	176
T92I	ACC-ATC	Thr-Ile	92	Y178N	TAC-AAC	Tyr-Asn	178
T94I	ACC-ATC	Thr-Ile	94	Y178D	TAC-GAC	Tyr-Asp	178
T97I	ACC-ATC	Thr-Ile	97	Y178C	TAC-TGC	Tyr-Cys	178
G101E	GGA-GAA	Gly-Glu	101	I179F	ATC-TTC	Ile-Phe	179
G101V	GGA-GTR	Gly-Val	101	P180A	CCC-GCC	Pro-Ala	180
V104I	GTC-ATC	Val-Ile	104	P180S	CCC-TCC	Pro-Ser	180
V104F	GTC-TTC	Val-Phe	104	E181K	GAG-AAG	Glu-Lys	181
G106R	GGG-AGG	Gly-Arg	106	G182S	GGC-AGC	Gly-Ser	182
G182V	GGC-GTC	Gly-Val	182	P291R	CCA-CGA	Pro-Arg	291
Q184P	CAG-CCG	Gln-Pro	184	A292T	GCG-ACG	Ala-Thr	292
C185R	TGC-CGC	Cys-Arg	185	A292E	GCG-GAG	Ala-Glu	292
S186P	TCG-CCG	Ser-Pro	186	A295V	GCC-GTC	Ala-Val	295
S186W	TCG-TGG	Ser-Trp	186	K296N	AAG-AAT	Lys-Asn	296
C187R	TGT-CGT	Cys-Arg	187	K296M	AAG-ATG	Lys-Met	296
C187G	TGT-GGT	Cys-Gly	187	K296E	AAG-GAG	Lys-Glu	296
C187Y	TGT-TAT	Cys-Tyr	187	S297R	AGC-AGA	Ser-Arg	297
G188R	GGA-AGA	Gly-Arg	188	A298D	GCC-GAC	Ala-Asp	298
G188E	GGA-GAA	Gly-Glu	188	A299T	GCC-AAC	Ala-Thr	299
D190N	GAC-AAC	Asp-Asn	190	V304D	GTC-GAC	Val-Asp	304
D190G	GAC-GGC	Asp-Gly	190	K311E	AAG-GAG	Lys-Glu	311
D190Y	GAC-TAC	Asp-Tyr	190	Q312Ter	CAG-TAG	Gln-Term	312
Y191C	TAC-TGC	Tyr-Cys	191	N315K	AAC-AAG	Asn-Lys	315
T193M	ACG-ATG	Thr-Met	193	T320N	ACC-AAC	Thr-Asn	320
N200K	AAC-AAG	Asn-Lys	200	L328P	CTG-CCG	Leu-Pro	328
F203S	TTT-TCT	Phe-Ser	203	A333V	GCC-GTC	Ala-Val	333
M207K	ATG-AAG	Met-Lys	207	T340M	ACG-ATG	Thr-Met	340
M207R	ATG-AGG	Met-Arg	207	E341K	GAG-AAG	Glu-Lys	341
V209M	GTG-ATG	Val-Met	209	E341Ter	GAG-TAG	Glu-Ter	341
V210F	GTC-TTC	Val-Phe	210	T2342M	ACG-ATG	Thr-Met	342
H211P	CAC-CCC	His-Pro	211	S343N	AGC-AAC	Ser-Asn	343
H211R	CAC-CGC	His-Arg	211	S343C	AGC-TGC	Ser-Cys	343
H211L	CAC-CTC	His-Leu	211	Q344P	CAG-CCG	Gln-Pro	344
I214N	ATC-AAC	Ile-Asn	214	Q344R	CAG-CGG	Gln-Arg	344
P215T	CCC-ACC	Pro-Thr	215	Q344Ter	CAG-TAG	Gln-Term	344
P215L	CCC-CTC	Pro-Leu	215	V345M	GTG-ATG	Val-Met	345
M216K	ATG-AAG	Met-Lys	216	V345L	GTG-CTG	Val-Leu	345
M216R	ATG-AGG	Met-Arg	216	V345G	GTG-GGG	Val-Gly	345
M216L	ATG-TTG	Met-Leu	216	V345L	GTG-TTG	Val-Leu	345
F220C	TTT-TGT	Phe-Cys	220	A346P	GCC-CCC	Ala-Pro	346
C222R	TGC-CGC	Cys-Arg	222	P347T	CCG-ACG	Pro-Thr	347
E249Ter	GAG-TAG	Glu-Term	249	P347Q	CCG-CAG	Pro-Gln	347
R252P	CGC-CCC	Arg-Pro	252	P347R	CCG-CGG	Pro-Arg	347
M253I	ATG-ATT	Met-Ile	253	P347L	CCG-CTG	Pro-Leu	347
P267R	CCC-CGC	Pro-Arg	267	P347A	CCG-GCG	Pro-Ala	347
P267L	CCC-CTC	Pro-Leu	267	P347S	CCG-TCG	Pro-Ser	347
S270R	AGC-AGA	Ser-Arg	270	Ter349Q	TAA-CAA	Term-Gln	349
G284S	GGT-AGT	Gly-Ser	284	Ter349E	TAA-GAA	Term-Glu	349
T289P	ACC-CCC	Thr-Pro	289				

a.a: amino acid.

## 2. Mechanisms of rhodopsin-related retinal disorders

Since the first rhodopsin mutation was identified in RP patients (Dryja et al., 1990), tremendous progress has been made toward understanding the mechanisms of retinal degeneration arising from rhodopsin mutations. Rhodopsin mutants exhibit a range of deficiencies in the 11-*cis*-retinaldehyde interaction (Liu et al., 1996; Iannaccone et al., 2006; Gragg and Park, 2018). There are two classes of rhodopsin mutations that have been designated based on their ability to bind the chromophore 11-*cis*-retinaldehyde when they are expressed in cultured cells (Kaushal and Khorana, 1994; Woods and Pfeffer, 2020). Class I mutants can reconstitute with 11-*cis*-retinaldehyde to form normal rhodopsin and are transported to the cell surface. Class II mutants are localized in the ER and cannot reconstitute with 11-*cis*-retinaldehyde to form functional rhodopsin, or binds 11-*cis*-retinaldehyde poorly. Rhodopsin mutations may cause protein misfolding and ER retention, mistrafficking, altered post-translational modifications and reduced stability, and constitutive action, which lead to photoreceptor death or dysfunction through divergent mechanisms. Dominant rhodopsin mutations with known features have been categorized into seven groups (Athanasiou et al., 2018). The following are the pathogenic mechanisms for representative rhodopsin mutations from four different groups whose mechanisms have been relatively well studied, including protein misfolding and ER retention (P23H), altered post-translational modifications and reduced stability (T17M), mistrafficking (Q344ter), and constitutive activation (G90D). More systemic description of these mechanisms can be found in a previous review (Athanasiou et al., 2018).

### 2.1. P23H

P23H, the first mutation reported in ADRP (Dryja et al., 1991), is the most common mutation found in rhodopsin in the United States (Wu et al., 2019; Woods and Pfeffer, 2020). P23H rhodopsin is a typical example of a class II mutation.

The pathogenesis of the P23H mutation has been extensively studied in a variety of animal models and *in vitro* cultured cells. The P23H mutation affects the adjacent H-bonding network in the chromophore binding region critical for the activity of the chromophore in rhodopsin expressed cultured HEK293 cells (Woods and Pfeffer, 2020). Additionally, it also alters the overall structure and activity of rhodopsin (Woods and Pfeffer, 2020). The monomer of mutant P23H is not functional, and it is unstable by itself and tends to adopt a specific homodimer arrangement (Woods and Pfeffer, 2020). P23H also exerts a destructive effect on disk membranes—likely through a homodimerization process—even at very low concentrations (Woods and Pfeffer, 2020). Another proposed possible pathogenic mechanism is related to activation of the UPR resulting from misfolding of the mutant protein as demonstrated in culture cells and transgenic mice (Frederick et al., 2001; Lin et al., 2007). UPR-mediated endoplasmic reticulum stress (ERS) triggers Ca^2+^ release from the ER, leading to activation of calpains and caspase-12 (Kitamura, 2008; Choudhury et al., 2013). Activated calpain can cleave the mitochondrial protein AIF (apoptosis-inducing factor), promoting AIF exit from the mitochondria through a pore formed by BAX and translocation into the nucleus, where AIF recruits cyclophilin A for chromatin condensation and fragmentation (Candé et al., 2004; Moubarak et al., 2007). AIF has been demonstrated to be present in the nuclei of most dying photoreceptor cells in P23H transgenic and knock-in mouse models (Comitato et al., 2016, 2020). Blocking calpain activity effectively protects the retina from degeneration in P23H knock-in mice (Comitato et al., 2020), suggesting calpain activation as one of the major causes of P23H mutant rhodopsin-induced photoreceptor degeneration.

### 2.2. T17M

The T17M mutation in *RHO* results in methionine replacing threonine at position 17 (Choudhury et al., 2013). This mutation is another class II mutantion that causes rhodopsin protein msifolding (Krebs et al., 2010), as described above. The mutation affects the binding of opsin proteins to 11-*cis*-retinaldehyde, resulting in ADRP (Li et al., 1998; Mendes et al., 2005; Krebs et al., 2010; Choudhury et al., 2013). The T17M rhodopsin mutant expressed in cultured cells is abnormally mislocalized in the endoplasmic reticulum (Li et al., 1998; Krebs et al., 2010; Jiang et al., 2014), with no colocalization with the Golgi apparatus as normal rhodopsin (Deretic and Papermaster, 1991), which may activate UPR and upregulating ERS-related proteins, such as BIP, GRP94, CHOP, peIF-2a/eIF-2a, and activating ATF-6a (Baumeister et al., 2005; Wang et al., 2009). Upregulation of ERS-related genes has been documented in the retina of T17M transgenic mice (Kunte et al., 2012). *In vitro* evidence suggests the mutant protein is unstable and susceptible to degradation by the proteasome system (Jiang et al., 2014). The demise of photoreceptors caused by T17M is partially attributed to the activation of caspase-7. Ablation of the gene encoding caspase-7 protects photoreceptors in T17M transgenic mice, likely through UPR reprogramming and inhibition of TRAF2-JNK apoptosis (Choudhury et al., 2013).

ROS are also suggested to play an important role in T17M-related retinal degeneration. ROS are the byproducts of aerobic metabolism, including oxygen ions, peroxides, and oxygen-containing free radicals (Jiang et al., 2014). High levels of ROS may damage lipids, proteins, and nucleic acids and affect the functions of organelles (Halliwell, 2011). Mitochondria are the main source of ROS. Increased ROS levels have been observed in cells expressing mutant T17M rhodopsin (Jiang et al., 2014), as ERS promotes chaperone activities that require more energy. Treatment of cells expressing T17M rhodopsin with ROS scavengers reduces cell death.

### 2.3. G90D

The G90D mutation, which is due to the substitution of aspartic acid for glycine at position 90 in rhodopsin, destabilizes a crucial ionic bond between E113 and K296 (Singhal et al., 2013; Silverman et al., 2020). *In vitro* data suggest that the G90D mutant belongs to a group of constitutively active mutants (including K29E) that can activate transducin in the dark (Rao et al., 1994; Robinson et al., 1994; Toledo et al., 2011; Park, 2014), resulting in a light-adapted state and the desensitization of rod photoreceptor cells in the dark (Sieving et al., 2001; Jin et al., 2003; Naash et al., 2004; Dizhoor et al., 2008). The mutation of neutral G to charged D alters the water-mediated H-bond network at the Schiff base region of the chromophore and the central transmembrane region, which may cause slow binding of the chromophore during pigment regeneration and constitutive activation of transducin (Rao et al., 1994; Gross et al., 2003). Persistent rhodopsin activation can cause retinal degeneration in both a transducin-dependent manner and a transducin-independent manner (Hao et al., 2002). Earlier studies showed that mutation of this gene causes congenital night blindness (Dizhoor et al., 2008). Patients with the G90D mutation have shown decreased light sensitivity of rod cells and night vision dysfunction. Most patients with the G90D mutation express normal amounts of rhodopsin, and the structure of the rods is well preserved (Sieving et al., 1995), while others exhibit the typical RP manifestation, in which the loss of rods is accompanied by the subsequent death of the cone cells and blindness (Berson, 1993). A cohort study comprised of 15 patients showed that 20 and 53.3% of patients with the G90D mutation displayed CSNB and classic RP, respectively, with no obvious sex differences (Kobal et al., 2021). Recently, slow retinal degeneration was also found in homozygous G90D transgenic mice (Colozo et al., 2020). From this point of view, the human G90D mutation may be right at the boundary between dysfunction and degenerative disease, providing an opportunity to further explore the mechanism by which the spontaneous activation of the visual transduction cascade leads to rod structure damage and cell death (Dizhoor et al., 2008).

### 2.4. Q344ter

The Q344ter mutation in rhodopsin causes the glutamine-encoding codon 344 to be replaced by a stop codon, resulting in early termination of the polypeptide and loss of the signature C-terminal motif sequence QVAPA. Rhodopsin is synthesized in the rough ER of the inner segment, processed by the Golgi apparatus, and then transported across the connecting cilium to the outer segment where phototransduction occurs. The C-terminal motif of rhodopsin is sufficient and necessary for rhodopsin to be correctly transported to outer segments. It has been shown that the carboxy-terminal cytoplasmic tail of the rhodopsin protein is involved in the post-Golgi transport of rhodopsin (Sung and Tai, 1999). The Q334Ter mutant expressed in transgenic animal models is mislocalized to the plasma membrane of the inner segments and subsequently translocated to the lysosome for degradation (Ropelewski and Imanishi, 2019). Degradation of mislocalized mutant rhodopsin causes disruption of plasma membrane protein homeostasis and downregulation of the sodium-potassium ATPase α-subunit (NKA). Compromised NKAα function is sufficient to cause shortening and loss of rod outer segments, which may underpin the mechanism of retinal degeneration related to the Q334Ter mutation (Ropelewski and Imanishi, 2019). A recent transcriptomic analysis of retinas from Q334Ter knock-in mice revealed alterations in the expression of chromatin complex genes such as histone genes (Bales et al., 2018). A more recent study showed that the upregulation of proinflammatory cytokines and pathways is involved the pathogenesis of retinal degeneration in this knock-in mouse model (Hollingsworth et al., 2021).

**Figure 1:**
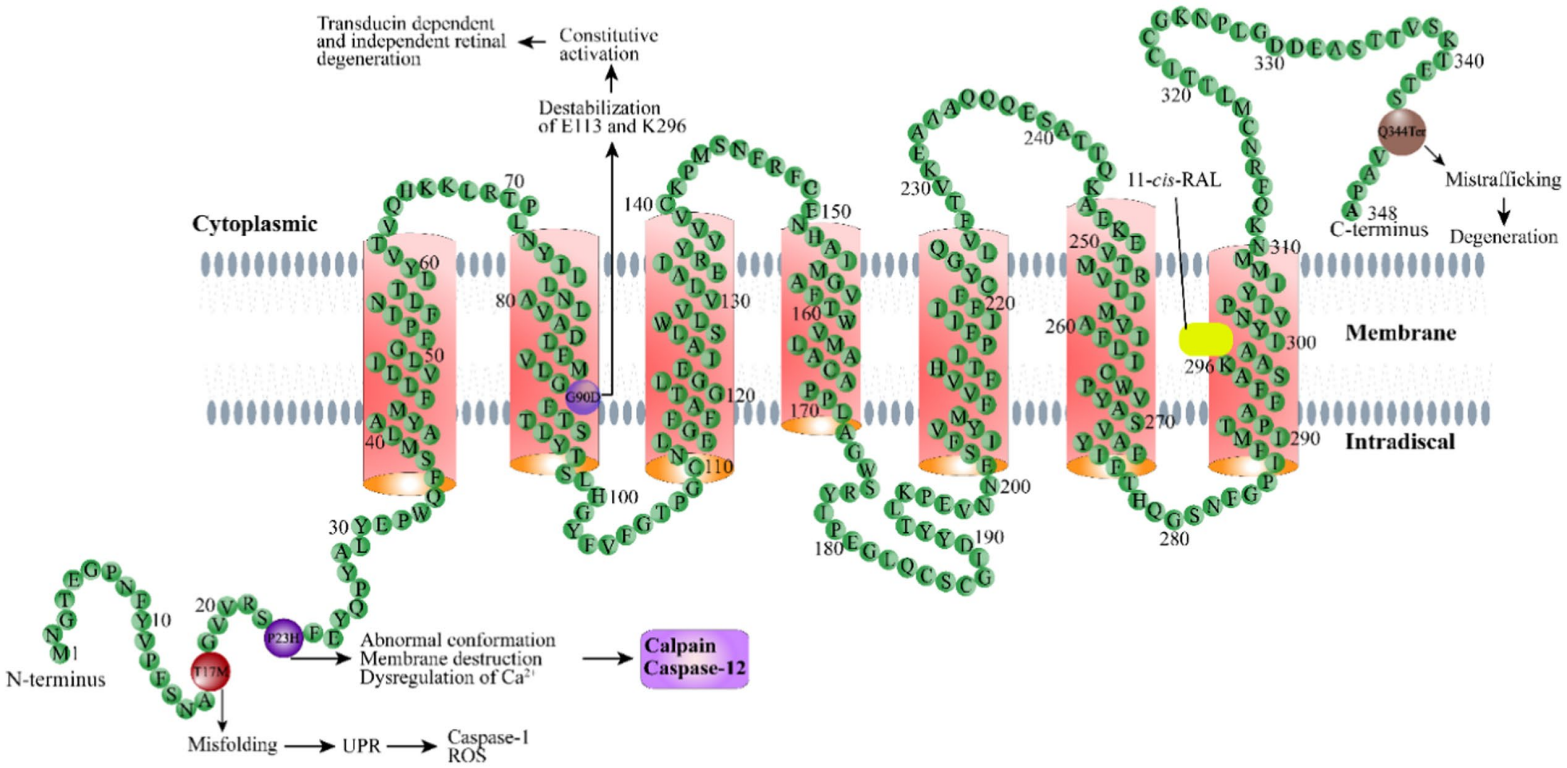
Schematic illustration of the rhodopsin structure. Rhodopsin is a G protein-coupled receptor with seven typical transmembrane domains. Representative rhodopsin mutations, including T17M, P23H, G90D, and Q344Ter, as well as the disease mechanisms related to these mutations, are shown. 11-*cis*-RAL: 11-*cis*-retinaldehyde.

These mechanisms represent a summary of representative pathogenesis related to rhodopsin mutations (Figure 1). Information for the mechanisms associated with other types of rhodopsin mutation can be found in several great earlier reviews (Athanasiou et al., 2018). Given the complexity of the interaction between all physiological processes and the wide spectrum of rhodopsin mutations, as well as the unique genetic background of each individual, we are still far from a complete understanding of these pathogenic mechanisms.

## 3. Strategies for therapy of rhodopsin-related retinal disorders

**Figure 2:**
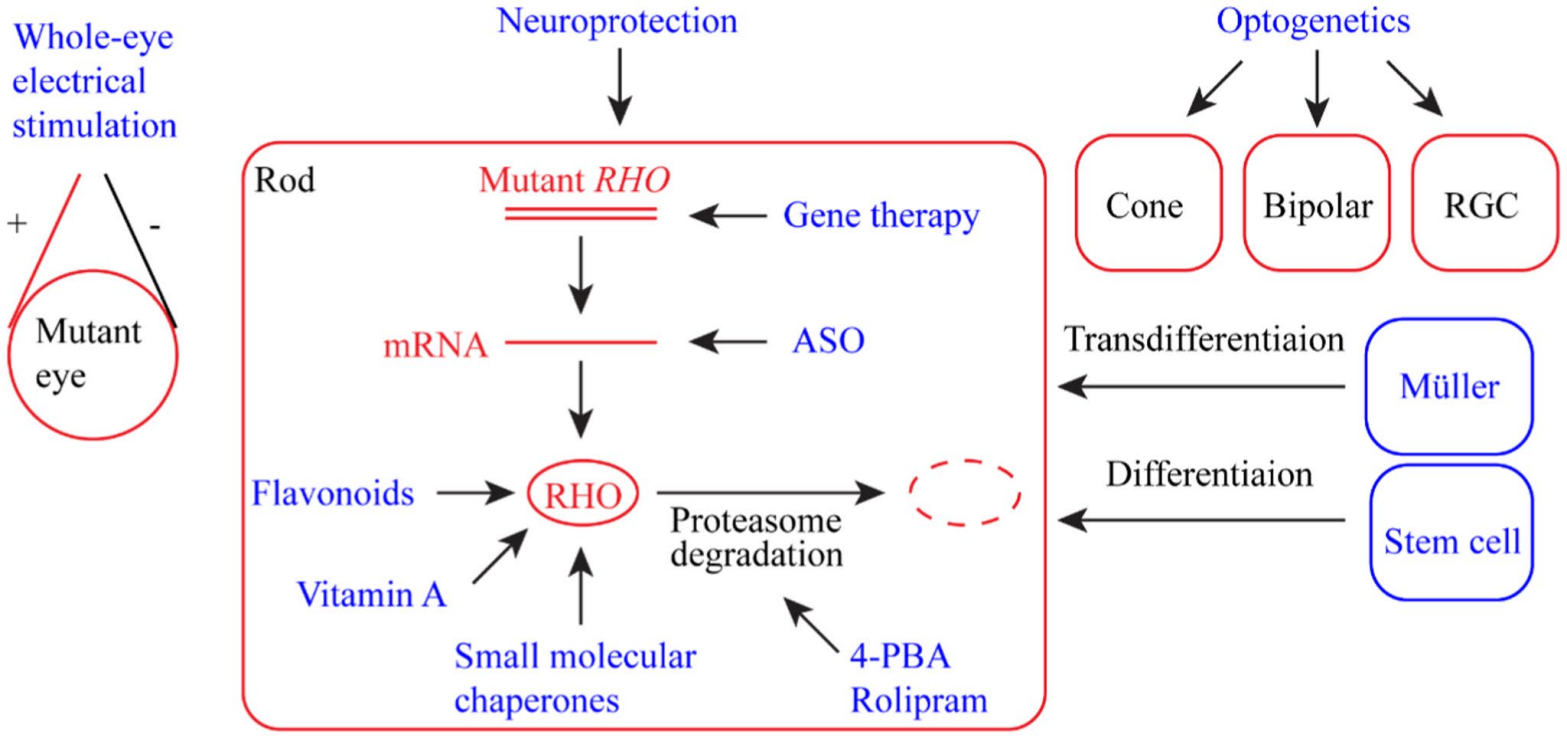
Summary of therapeutic strategies for retinal dystrophy (RD) associated with rhodopsin mutations. The strategies include whole-eye electric stimulation, pharmacological therapy (flavonoids, vitamin A, small molecular chaperones, 4-PBA (4-phenyl butyric acid), and rolipram), ASO (antisense oligonucleotide) therapy, gene therapy, neuroprotection, optogenetic therapy, and stem cell therapy (including photoreceptor regeneration from Müller cells).

RP-related rhodopsin mutations are highly heterogeneous, with over 200 mutations identified (Loewen et al., 2020). Conventional treatments have little effect in terms of the cure and prevention of retinal degeneration as a result of the wide spectrum of rhodopsin mutations. In the past two decades, enormous efforts have been directed toward developing innovative treatment methods or drugs to protect vision from retinal degeneration caused by RP, particularly rhodopsin mutations. Here, we summarize the developed strategies that potentially delay the degeneration of retinal photoreceptor cells and preserve vision (Figure 2). Some of these strategies, such as gene therapy and stem cell therapy, can be applied to RP caused by mutations in genes other than *RHO*.

### 3.1. Whole-eye electrical stimulation

Electrical stimulation therapy (EST), as a classic physical therapy, can improve muscle and nerve function. As early as the 19th century, the rehabilitative effects of electrical stimulation on the eyes were observed (Dor, 1873). The administration of low-level electric current by various approaches has been demonstrated to improve visual function, validating the feasibility of this therapeutic strategy in the treatment of eye diseases (Hanif et al., 2016). Using methods such as subretinal implants or transcorneal electrical stimulation (TES), low-level electrical stimulation of the eye has been shown to have neuroprotective effects on retinal degeneration in humans and animal subjects (Ciavatta et al., 2013; Hanif et al., 2016; Yu et al., 2020).

*RHO* P23H transgenic rats receiving 30 min of low-level electrical stimulation at a frequency of twice a week (4 μA at 5 Hz; *n* = 10) from 4 to 24 weeks of age exhibited significant improvements in visual function, as exemplified by better responses in the electroretinography (ERG) test (Hanif et al., 2016). The underlying mechanism could be due to increased expression of neuroprotective factors, such as ciliary nerve trophic factor (CNTF) and brain-derived neurotrophic factor (BDNF), in Müller cells (MCs; Ferrari et al., 2011; Gall et al., 2011; Hanif et al., 2016). Transcorneal electrical stimulation therapy in 21 patients with RP showed that TES might cause trivial symptoms such as foreign body sensation, burning, and itching, which, however, were mild and controllable (Demir et al., 2022). In the future, EST can be combined with other treatment methods to increase its efficacy.

### 3.2. Pharmacological therapy

Pharmacological compounds are often used to alleviate or treat various types of RP due to their various beneficial factors, such as their wide variety and easy availability. Several types of pharmacological compounds have been experimentally and clinically tested to treat retinal dystrophies caused by rhodopsin mutations.

#### 3.2.1. Flavonoids

GPCR activities can be modulated by exogenous or endogenous molecules. Flavonoids are functional modifiers that can alter conformation and strengthen the expression of visual receptors (Ortega et al., 2019, 2021). Flavonoids are a group of yellow pigments with flavonoids (2-phenylchromones) as the parent compound, including isomers of flavonoids and their hydrogenated reduction products. Flavonoids are commonly found in fruits and vegetables. Due to their antioxidant, anti-inflammatory, and anti-apoptotic properties, flavonoids have been documented to improve vision in various ophthalmic diseases (Majumdar and Srirangam, 2010; Ortega et al., 2019). They also help stabilize ligand-free opsin, which promotes retinal degeneration when present in excess in the retina (Ortega et al., 2021). The binding of flavonoids to the P23H mutant rhodopsin changes the protein conformation and partially restores its intracellular transport, which slows photoreceptor degeneration in a mouse model (Ortega et al., 2019, 2022). The flavonoid quercetin can allosterically modulate opsin regenerated with 9-*cis*-retinaldehyde and enhance the stability and conformational properties of the G90V mutant (Herrera-Hernandez et al., 2017). Therefore, flavonoids can potentially be used as primary compounds to design nonretinoids for the treatment of retinal degeneration associated with rhodopsin mutations (Herrera-Hernandez et al., 2017; Ortega et al., 2019, 2022).

#### 3.2.2. Vitamin A

Retinaldehyde (or retinal), also known as vitamin A aldehyde, is a derivative of retinol after oxidation or is produced by the oxidative cleavage of β-carotene. Retinaldehyde is a prosthetic group of rhodopsin. Retinaldehyde is mainly converted from vitamin A supplemented from the blood. One study showed that vitamin A supplementation helps preserve rod photoreceptors and visual function in a T17M (class II mutation) transgenic mouse (Li et al., 1998) but has little beneficial effect on P347S (class I mutation) mutant mice (Li et al., 1998). More importantly, a recent study showed that the D190N transgenic mice on the vitamin A diet exhibited higher levels of autofluorescence and lipofuscin metabolites, raising concerns about the potential detrimental effect of vitamin A supplementation on the retina expressing D190N (Cui et al., 2022). As the vitamin A method depends on the context of mutations, when this therapeutic method is translated from the laboratory to clinical treatment, the patients should be genotyped for the mutation in rhodopsin to determine whether this treatment method should be initiated.

#### 3.2.3. 4-PBA (4-phenyl butyric acid) and rolipram

Misfolded proteins caused by the P23H mutation activate ERS responses, which may trigger imbalanced activation of autophagy relative to the proteasome (Qiu et al., 2019), leading to activation of protein degradation and cell death pathways in photoreceptor cells. The autophagy and ubiquitin–proteasome degradation pathways are two important methods of intracellular quality control and recycling that are responsible for cellular homeostasis in eukaryotes. The misfolded proteins caused by genetic mutations may lead to the persistent activation of the autophagy pathway. The autophagy pathway cannot completely deal with misfolded proteins, which may lead to secondary proteasome degradation and proteasome deficiency or decreased proteasome activity. A recent study showed that reducing ERS-induced autophagy activation while simultaneously increasing proteasome activity improves photoreceptor survival (Qiu et al., 2019), which suggests a potential new therapeutic strategy for the treatment of ADRP caused by protein folding defects.

This idea has also been validated in experiments using 4-phenyl butyric acid (4-PBA) and rolipram. While 4-PBA is a chemical partner that improves protein folding and protein shuttling to the proteasome pathway, rolipram is a selective phosphodiesterase-4 inhibitor that can directly increase proteasome activity (Zeitlin et al., 2002; Malo et al., 2013; Qiu et al., 2019). Both compounds have shown decreased ERS metabolism, decreased activation of the cell death pathway, and improvements in terms of photoreceptor morphology and visual function in a P23H mouse model (Powers et al., 2009; Qiu et al., 2019). Concomitantly, the transcription levels of the proapoptotic genes Fas and caspase 8 are downregulated (Yao J. et al., 2018; Qiu et al., 2019). Decreased phosphorylation of MLKL and RIPK3, which are two markers of necroptosis activation, was also observed in 4-PBA-treated mice. Therefore, 4-PBA rescues photoreceptors by suppressing both apoptotic and necroptotic cell death. In addition, a recent study using P23H knock-in mice suggests that 4-PBA may protect photoreceptors by modulating the mitochondrial function through epigenetic regulation (Ozawa et al., 2022). Despite the encouraging evidence in the preclinical animal model, the application of these compounds to treat inherited retinal diseases (IRDs) should be performed with caution, as neuroprotective therapies for IRDs failed to yield positive results in a clinical trial (Birch et al., 2016). The main reason could be that blocking one death pathway may activate other potential death pathways (Qiu et al., 2019).

#### 3.2.4. SRD005825, YC-001, and TUDCA

To address the pathogenesis caused by protein misfolding, pharmacological compounds, such as small molecule chaperones, have been developed to help stabilize the protein structure. This is another strategy for the treatment of RP. SRD005825, YC-001, and TUDCA are three representative compounds that are used in this regard. While SRD005825 and YC-001 are two recently developed compounds, TUDCA is a natural compound that has been extensively studied.

##### 3.2.4.1. SRD005825

SRD005825, also known as SHP630, is an analog of 9-*cis*-retinaldehyde but does not covalently bind to opsin as a chromophore (Ahmed et al., 2019). *In vitro* assays showed that SRD005825 competes for 9-*cis*-retinaldehyde binding for purified rhodopsin. SRD005825 facilitates the reconstitution of mutant rhodopsin proteins and promotes T17M mutant rhodopsin translocation to the plasma membrane (Ahmed et al., 2019). Treatment with SRD005825 significantly slows the rate of rapid retinal degeneration in T17M mutant mice. SRD005825 also induces mutant rhodopsin to adopt a normal conformation and improves the light response following treatment in T17M mice. SRD005825 is a promising candidate for the treatment of RP caused by misfolded mutant rhodopsin.

##### 3.2.4.2. YC-001

YC-001 is a novel nonretinoid pharmacological chaperone of rod photoreceptor opsin (Chen et al., 2018). Compared with 9-*cis*-retinaldehyde, YC-001 exhibits micromolar potency and greater efficacy but with a lower cytotoxicity. The chaperone activity of YC-001 is demonstrated by its ability to rescue multiple rhodopsin mutants in mammalian cells. By binding to rhodopsin, YC-001 antagonizes opsin signaling in a noncompetitive manner. YC-001 regulates the synthesis of the P23H mutant protein and stabilizes its structure in the ROS disk upon bleaching by light. Additionally, YC-001 is able to rescue the transport of other class II rhodopsin mutants, such as G106R, D190N, and P267L, but not P53R or C110Y, suggesting variation in folding defects among different class II mutants. Of importance, the metabolism of YC-001 in mice is significantly different from that in humans. Hence, the application of YC-001 for the treatment of human RP requires additional testing and evaluation (Chen et al., 2018).

##### 3.2.4.3. TUDCA

TUDCA is a natural compound found in bear bile that has been used in Asia for over 3,000 years to treat visual disorders (Boatright et al., 2006). TUDCA protects neurons from apoptosis in neurodegenerative diseases (Duan et al., 2002; Keene et al., 2002), possibly by reducing ERS and inflammatory responses (Beuers et al., 1992; Özcan et al., 2006). TUDCA treatment is capable of preserving cone and rod structure and function and the connections between photoreceptor cells and postsynaptic neurons in a P23H rat model (Fernandez-Sanchez et al., 2011, 2015). In parallel, TUDCA reduces the number of microglia in P23H transgenic rats and prevents their activation (Noailles et al., 2014). Additionally, TUDCA reduces the presence of macrophages. Thus, the neuroprotective effect of TUDCA is mediated by its anti-inflammatory properties. Although TUDCA has proven to be effective in the protection of retinal neurons in P23H rats, its application in clinical trials is limited by the fact that high systemic concentrations are required to achieve local neuroprotective effects. Technically, it is difficult to maintain a high concentration of the drug in the eye for the long term. A persistently high concentration of the drug can be achieved by systemic high-concentration administration or frequent intraocular injections, which, however, may cause pharmacological toxicity and physical damage, respectively (Fernández-Sánchez et al., 2017). One potential solution could be loading the drug into a biodegradable microsphere that enables a slow and sustained release (Fernández-Sánchez et al., 2017); however, the feasibility of this concept requires further testing.

### 3.3. Antisense oligonucleotide therapy

Antisense-mediated gene suppression was first reported in 1978 (Zamecnik and Stephenson, 1978). In recent years, antisense oligonucleotides (ASOs) have emerged as a potential strategy for the treatment of inherited retinal diseases (Collin and Garanto, 2017). ASOs are small DNA or RNA molecules that are complementary to their target mRNAs. Therapeutic ASOs can be chemically synthesized oligonucleotides 18–30 nucleotides in length. The binding of ASOs to targeted RNAs promotes RNA fragmentation and degradation. They may also inhibit the expression of target RNA by blocking the translation machinery. One study showed that intravitreal administration of second-generation ASOs effectively and specifically reduces the level of allele-specific mutant rhodopsin in a transgenic rat that expresses a murine P23H rhodopsin gene (Murray et al., 2015). In this study, the treated eyes also exhibited improved photoreceptor morphologies, function, and cell survival. ASOs have several apparent advantages over other gene-silencing agents (Murray et al., 2015), such as high selectivity for alleles with single-base mutations and simple delivery in a water-based formulation (Østergaard et al., 2013). A first-generation ASO for the treatment of cytomegalovirus (CMV) retinitis has been approved by the FDA (Group, 2002; Jabs and Griffiths, 2002). The half-life of second-generation ASOs is notably longer than that of first-generation ASOs. Thus, the treatment cycle will also be longer without the need for frequent injections (Murray et al., 2015). These advantages make ASOs great candidates for the treatment of retinal degeneration caused by rhodopsin mutations.

### 3.4. Gene therapy

Gene therapy enables the addition of exogenous genes to correct pathogenic symptoms. Depending on the mutation type, several strategies have been developed to treat RP. Gene augmentation is a simple and straightforward strategy featuring the transfer and expression of wild-type exogenous genes into host cells. This approach has proven to be successful in the treatment of autosomal recessive RP both experimentally and clinically (Kumar et al., 2016; Bennett, 2017). However, this strategy had limited success in treating ADRP. Although gene augmentation may improve pathogenic symptoms by diluting mutant proteins, the gain-of-function or dominant-negative mutant proteins that remain within the cells will still exert their toxic effects. Therefore, researchers are striving to develop alternative strategies to treat ADRP, particularly rhodopsin-associated RP, which accounts for over 25% of all ADRP cases (Dryja et al., 1991; Sullivan et al., 2013).

#### 3.4.1. Mutation-independent strategies

One of these alternative strategies is to nonselectively knock down both the mutant and WT *RHO* with the concomitant expression of resistant WT *RHO* as a replacement. This resistant WT *RHO* is achieved by using synonymous codons at the target site. Ribozyme, zinc-finger–based artificial transcription factors, and RNAi have been utilized to suppress the expression of endogenous target genes (LaVail et al., 2000; Jiang et al., 2011; Mussolino et al., 2011). A recent study showed that a single vector expressing both shRNA and a human *RHO* replacement cDNA made resistant to RNA interference was successful in treating a naturally occurring canine model of RHO-ADRP with the T4R mutation (Cideciyan et al., 2018). The highly potent shRNA nearly completely suppressed the endogenous canine *RHO* RNA, and the replacement cDNA expressed 30% of the normal RHO protein. Treatment of P23H transgenic mice using the same shRNA and shRNA-resistant human slowed retinal degeneration during the 9-month study period (Ahmed et al., 2023). An apparent advantage of this mutation-independent strategy is that one single construct can be applied to the treatment of ADRP caused by different rhodopsin mutations, with over 200 identified, which will be much more cost-friendly for potential patients. Several clinical trials using this treatment strategy have been launched and planned to be initiated (Meng et al., 2020).

Nevertheless, this strategy is not free of disadvantages, as knockdown of endogenous rhodopsin RNA with siRNA may cause cell toxicity (Grimm et al., 2006). Moreover, the toxicity of overexpressed rhodopsin in the photoreceptors should also be taken into account. The off-target effect of shRNA should be another concern to be considered. To circumvent these disadvantages of shRNA, a novel approach using artificial mirtrons has been tested. Mirtrons are derived from spliced-out introns that regulate gene expression in a way similar to classic microRNAs (miRNAs; Curtis et al., 2012). A strategy using mirtron-based knockdown in combination with gene replacement has been shown to reduce disease severity in a P23H knock-in mouse model (Orlans et al., 2021). An alternative strategy is using a CRISPR-mediated system to ablate the endogenous *RHO* gene in combination with the optimized *RHO* replacement. This strategy has achieved promising results in P23H and D190N mouse models (Tsai et al., 2018). Similarly, EDIT-3, developed to target human *RHO*-assoicated ADRP, is currently undergoing preclinical trials (Meng et al., 2020).

#### 3.4.2. Gene editing

Another concern related to the knockdown/replacement strategy is the duration of its efficacy. The recently emerging CRISPR/CAS9 technique is a solution to this concern. CRISPR/CAS9-mediated gene editing can correct the mutation and restore the normal function of the targeted gene (Rasoulinejad and Maroufi, 2021). A report showed that this technique was capable of correcting ~45% of the mutant allele at the DNA level in a P23H mouse model, significantly delaying the progression of photoreceptor degeneration in the treated area (Li et al., 2018). Allele-specific editing also effectively ameliorates dominant RP in an *RHO* P347S transgenic mouse model (Patrizi et al., 2021). A recently developed CRISPR-mediated DNA base editors enable corrections of pathogenic single nucleotide variants (SNVs) in rhodopsin (Komor et al., 2016), whereas the CRISPR-based primer editing system is even more versatile, potentially installing any combination of point mutations, small insertions or small deletions (Anzalone et al., 2019). Targeting of the CAS9 protein to the desired genomic position requires the presence of a construct-specific protospacer adjacent motif (PAM) and a guide RNA molecule. A systemic survey of 247 reported pathogenic *RHO* variants for suitable PAM sites for currently available base editors showed that 55% of those SNVs are editable with base editors and only 32% of them harbor PAM sites (Kaukonen et al., 2022). CAS9 variants have been developed to overcome the restriction of PAMs (Miller et al., 2020; Huang et al., 2023), which may greatly expand the use of base editors. The safety of this allele-specific editing and base editing strategies requires further research for validation.

### 3.5. Neuroprotection by trophic factors

Trophic factors are secreted small proteins that regulate cell proliferation, maturation, and viability (Snider and Johnson, 1989; von Bartheld, 1998). Some trophic factors have been documented to effectively delay retinal degeneration in various animal models (Faktorovich et al., 1990; LaVail et al., 1992; Unoki and LaVail, 1994; Green et al., 2001; Miyazaki et al., 2003; Buch et al., 2006). Basic fibroblast factor (bFGF) was the first factor to demonstrate neuroprotective effects on degenerative photoreceptors in light-damaged rats, and RCS rats suffer from a mutation in the MERKT gene (Faktorovich et al., 1990). Since then, more neurotrophic factors, including brain-derived neurotrophic factor (BDNF; LaVail et al., 1992), ciliary neurotrophic factor (CNTF; Unoki and LaVail, 1994), glial cell line-derived neurotrophic factor (GDNF; Buch et al., 2006), and pigment epithelium-derived factor (PEDF; Miyazaki et al., 2003), have been found to effectively counter retinal degeneration.

As neurotrophic factors are proteins, they are susceptible to degradation by proteases. Consequently, they have a relatively short half-life and can only provide short-term protection. Viral vector-mediated delivery of genes encoding neurotrophic factors has been shown to achieve sustained expression of these factors and offer long-term protection. One study showed that FGF-15 and FGF-18 expressed from the recombinant AAV virus notably delayed retinal degeneration caused by transgenic rats expressing a P23H or Q334ter rhodopsin mutation (Green et al., 2001). FGF-15 and FGF-18 are two members of the fibroblast factor family. In addition, AAV-mediated expression of CNTF prolongs photoreceptor survival in mutant rhodopsin mice (Liang et al., 2001). A recent study showed that treatment of P23H rats using neurotrophic factors in combination with suppression of microglia by minocycline achieved better results than using neurotrophic factors alone. Thus, neurotrophic factors are promising candidates for the treatment of retinal degeneration related to rhodopsin mutations.

Although CNTF and PEDF have been shown to rescue photoreceptor morphologies and prolong photoreceptor survival in rodent models of retinal degeneration, they may suppress retinal function, as determined by ERG recoding (Liang et al., 2001; Miyazaki et al., 2003; Buch et al., 2006). Additionally, translation of this approach into clinical applications encountered a major setback, based on one clinical study showing that RP patients treated with CNTF released continuously from an intravitreal implant had a greater loss of total visual field sensitivity in the treated eyes than in the sham-treated eyes (Birch et al., 2016). The reason for the contradictory results between animal models and human patients is unknown. Therefore, the use of neurotrophic factors for treating RP requires further evaluation.

### 3.6. Optogenetic therapy

During the process of RP, the progressive death of rod photoreceptors is followed by cone cell death. After photoreceptor cells die in advanced RP, the structure and function of the remaining cells, such as retinal ganglion cells and bipolar cells, remain intact. Therefore, it is possible to convert these remaining light-insensitive cells into photosensitive cells using an optogenetic therapy approach, thereby partially restoring vision (Pan et al., 2015). Channelrhodopsin 2 (ChR2) is a widely used optogenetic protein for this purpose. ChR2, originally cloned from *Chlamydomonas reinhardtii* (Nagel et al., 2003), is a direct light-gated cation ion channel that opens rapidly upon the absorption of photons and depolarizes the cell membrane (Nagel et al., 2002; Busskamp et al., 2010; Mutter et al., 2014). Restoration of vision using an optogenetic strategy was achieved in preclinical mouse and rat models of RP (Bi et al., 2006; Tomita et al., 2007, 2010). In addition to optogenetic proteins, chemical photoswitchs, such as DENAQ, AAQ and diethylamino-azo-diethylamino (DAD), have been shown to restore retinal responses to light in mice with degenerated photoreceptors (Polosukhina et al., 2012; Tochitsky et al., 2014; Laprell et al., 2017). A recent clinical study showed that a blind patient partially recovered visual function after optogenetic therapy using the channelrhodopsin protein ChrimsonR fused to the red fluorescent protein tdTomato (Sahel et al., 2021). The patient recovered to the extent that he could recognize the location of objects and reach out to locate them with the help of goggle-assisted-light stimulation. As optogenetic therapy is a gene and mutation-independent approach, it is conceivable that optogenetic therapy is quite promising to restore partial vision for advanced RP patients with rhodopsin mutations.

### 3.7. Stem cell therapy

With the improvement of our understanding of stem cells, stem cell therapy has emerged as a promising treatment method for RP, particularly RP at an advanced stage when most photoreceptors are lost and gene therapy is difficult. There are two major strategies for treating RP using stem cell therapy: (1) the transplantation of exogenous stem cells into the retina and the induction of the differentiation of the cells into desired cell types and (2) the induction of transdifferentiation of MCs into other types of neural cells.

#### 3.7.1. Transplantation of exogenous stem cells

As early as 1988, retinal pigment epithelium (RPE) transplantation was tested for the treatment of RCS rats with defective RPEs (Li and Turner, 1988). In addition to RPE, transplantation of stem cells or cells differentiated from stem cells has been explored to protect or substitute for neurons in retinal disorders, particularly for late-stage retinal degeneration. Stem cells for treatment purposes include embryonic stem cells (Lund et al., 2006; Idelson et al., 2009; Lu et al., 2009), induced and reprogrammed stem cells (Sun et al., 2015), and adult tissue stem cells (Kicic et al., 2003; Arnhold et al., 2007; Xiong et al., 2011). Embryonic stem cells are derived from a developing embryo. However, the source of embryonic stem cells, in particular human embryonic stems, is limited and may raise ethical concerns (Ikelle et al., 2020). With the advent of induced pluripotent stem cells (iPSCs) that are reprogrammed from adult somatic cells by the transfection of defined critical transcription factors, such as SOX2, Klf4, c-Myc, and Oct4 (Takahashi and Yamanaka, 2006), the supply of stem cells is essentially unlimited without ethical restriction. Nevertheless, the use of iPSCs is limited, as they tend to result in immune rejection and teratogenicity (Holan et al., 2021). Adult tissue stem cells include mesenchymal stem cells (MSCs) and neural stem cells (NSCs).

In the past decade, human adult bone marrow mesenchymal stem cells (BM-MSCs) have gained exclusive attention for the treatment of retinal degeneration due to their unique properties (Zaverucha-do-Valle et al., 2011; Tzameret et al., 2014; Weiss and Levy, 2020). BM-MSCs are easily expandable, with a broad differentiation potential into other cell types (Ferrari et al., 1998; Kopen et al., 1999; Kicic et al., 2003; Vandervelde et al., 2005; Oh et al., 2008), such as neurons and astrocytes. They can be used for autologous transplantation. Thus, they are safer than embryonic stem cells. Moreover, autologous transplantation may promote survival and enhanced therapeutic effects. Mesenchymal stem cells can be introduced into the eyes *via* the intraocular injection of cell suspension and cell patches. Upon the *in vitro* stimulation of growth and differentiation factors, including FGF2, taurine, retinoic acid, and IGF-1, MSCs can differentiate into precursor photoreceptor cells depending on their surface markers, such as specific antigens (Jayaram et al., 2014). Once differentiated, the photoreceptor cells integrate into the photoreceptor layer in the degenerated retina, and they can improve retinal function and restore vision. BM-MSCs have been demonstrated to restore lost visual function in animal models with retinal dystrophies (Lu et al., 2010; Zaverucha-do-Valle et al., 2011). Subretinal transplantation of BM-MSCs has been demonstrated to rescue photoreceptors in rhodopsin knockout mice (Arnhold et al., 2007), suggesting the possibility of using BM-MSCs to treat human RP caused by rhodopsin dysfunction.

Currently, several limitations of stem therapy need to be overcome before it becomes a practical clinical therapeutic approach. A technical obstacle faced by stem cell therapy is how to homogeneously disperse the transplanted cells into the retina to cover a large area. Another limitation is that it is difficult for the transplanted cells to migrate and integrate into the existing neural network to function correctly, although a recent study showed that the degradation of the extracellular matrix with chondroitinase ABC promotes cell migration (Ding et al., 2019). Another major issue is that the induction rate of the differentiation of the transplanted cells into the target cells is relatively low. According to reports, one team successfully differentiated photoreceptor cells *in vitro* under EPO treatment conditions (Ding et al., 2019). Another group performed intravitreal bone marrow mesenchymal stem cell transplantation in three patients with advanced RP, with adverse effects observed in two of them after transplantation. Between 2 weeks and 3 months after transplantation, patients reported an improvement in their perception of light. Another patient was observed to have severe fibrous tissue proliferation, resulting in tractional retinal detachment (Satarian et al., 2017). Simple bone marrow MSC transplantation is currently imperfect, and animal studies are recommended before implementation of clinical trials (Satarian et al., 2017).

#### 3.7.2. Autologous stem cell induction therapy

In addition to transplantation of exogenous cells, emerging evidence shows that diseases due to photoreceptor cell loss can be treated by inducing the differentiation of MCs present in the eye into destination cells. MCs are the primary retinal glial cells with protrusions that span the entire thickness of the retina (Bringmann and Reichenbach, 2001). MCs in the human retina are thought to be dormant retinal precursor cells that can regenerate retinal neurons when the retinal tissue is damaged (Karl and Reh, 2010; Yu et al., 2014; Jorstad et al., 2017; Yao K. et al., 2018). Thus, awakening the regenerative potential of the retina is a promising way to repair degenerated retinas (Yu et al., 2014).

A recent study showed that the absence of Ephrin-A2/A3 promoted retinal regenerative potential of MCs in mice lacking rhodopsin (Zhu et al., 2021). The Ephrin family and its receptor, Eph, are the key regulators of CNS development, neural cell migration, and MC proliferation. Ephrin-A2/A3 and its receptor, Ephrin-A4, are both expressed in the retina, especially in MCs, but under physiological conditions, Ephrin signaling inhibits the neurogenic potential of MCs. The expression of Ephrin-A2/A3 and its receptor Ephrin-A4 increases with retinal maturation and proliferation, while the neurogenic potential of progenitor cells decreases, and Ephrin-A2/A3 is a negative regulator of MC proliferation and neurogenic potential. Controlling Ephrin-A2/A3 expression promotes the migration of proliferating cells to the photoreceptor cell layer for regeneration and the replacement of lost cells (Zhu et al., 2021). In *Rho^−/−^*/*Ephrin A2^−/−^*/*Ephrin A3^−/−^* triple knockout mice, significantly more MCs were detected in the inner nuclear layer than in *Rho* knockout mice, and proliferating MCs could also be detected to have migrated to the outer nuclear layer (Zhu et al., 2021).

## 4. Conclusion

Retinal dystrophy associated with rhodopsin mutations is an inherited disease with a pathogenesis that largely stems from cellular autophagy induced by abnormal retinoid binding to mutant proteins leading to cytotoxicity. A growing body of laboratory and clinical evidence suggests that the dysregulation of calcium homeostasis and unfolded protein responses play vital roles in retinal degeneration as independent or combined pathogenic mechanisms. The development of drugs targeting molecules these pathways may provide new therapeutic approaches for retinal degeneration. In recent decades, vitamin A supplementation has become the main therapy method; however, its use in clinical practice is limited because the effects do not significantly improve or delay the rate of retinal degeneration, especially in patients with advanced RP. Innovative therapy strategies, such as gene therapy (including gene editing, neuroprotection, and optogenetics) and stem cell therapy, are promising methods for the future treatment of RP. Greater efforts are needed from researchers and clinicians to facilitate the translation of recent research findings from the laboratory into clinical practice.

## Author contributions

FZ and TZ wrote the manuscript. TW and YZ made the figures. SD and HZ edited the manuscript. All authors contributed to the article and approved the submitted version.

## Funding

This work was supported by the grants from the National Natural Science Foundation of China (nos. 81770935 to HZ and 81800830 to SD), the Department of Science and Technology of Sichuan Province (no. 2023JDZH0002 to HZ), Young and Middle-aged Health Science and Technology Innovation Talent Training Project (Outstanding Young Persons) of Henan Province (no. YXKC2022025 to SD), Medical Science and Technology Project (the Key Project Jointly Built by the Province and the Ministry) of Henan Province (no. SBGJ202102167 to SD), and the Key Research and Development and Promotion Project (Science and Technology) program of Henan Province (no. 192102310077 to SD).

## Conflict of interest

The authors declare that the research was conducted in the absence of any commercial or financial relationships that could be construed as a potential conflict of interest.

## Publisher’s note

All claims expressed in this article are solely those of the authors and do not necessarily represent those of their affiliated organizations, or those of the publisher, the editors and the reviewers. Any product that may be evaluated in this article, or claim that may be made by its manufacturer, is not guaranteed or endorsed by the publisher.

## Footnotes

^1^ https://sph.uth.edu/retnet

^2^ https://www.hgmd.cf.ac.uk/ac/
